# Resistive Memory-Switching Behavior in Solution-Processed Trans, trans-1,4-bis-(2-(2-naphthyl)-2-(butoxycarbonyl)-vinyl) Benzene–PVA-Composite-Based Aryl Acrylate on ITO-Coated PET

**DOI:** 10.3390/polym16020218

**Published:** 2024-01-12

**Authors:** Rachana Kamath, Parantap Sarkar, Sindhoora Kaniyala Melanthota, Rajib Biswas, Nirmal Mazumder, Shounak De

**Affiliations:** 1 Department of Electronics and Communication Engineering, Manipal Institute of Technology, Manipal Academy of Higher Education, Manipal 576104, Karnataka, India; kamathrach@gmail.com; 2 Manipal Centre for Natural Sciences, Manipal Academy of Higher Education, Dr. T. M. A. Pai Planetarium Building, Madhav Nagar, Manipal 576104, Karnataka, India; parantaporgchem@gmail.com; 3 Department of Biophysics, Manipal School of Life Sciences, Manipal Academy of Higher Education, Manipal 576104, Karnataka, India; sindhoora.km@learner.manipal.edu (S.K.M.); nirmal.mazumder@manipal.edu (N.M.); 4 Department of Physics, Tezpur University, Tezpur 784028, Assam, India; rajib@tezu.ernet.in

**Keywords:** non-zero-crossing current–voltage characteristic, resistive switching behavior, capacitive and negative differential resistance (NDR) effects, Ohmic conduction, filamentary conduction

## Abstract

Resistive switching memories are among the emerging next-generation technologies that are possible candidates for in-memory and neuromorphic computing. In this report, resistive memory-switching behavior in solution-processed trans, trans-1,4-bis-(2-(2-naphthyl)-2-(butoxycarbonyl)-vinyl) benzene–PVA-composite-based aryl acrylate on an ITO-coated PET device was studied. A sandwich configuration was selected, with silver (Ag) serving as a top contact and trans, trans-1,4-bis-(2-(2-naphthyl)-2-(butoxycarbonyl)-vinyl) benzene–PVA-composite-based aryl acrylate and ITO-PET serving as a bottom contact. The current–voltage (I–V) characteristics showed hysteresis behavior and non-zero crossing owing to voltages sweeping from positive to negative and vice versa. The results showed non-zero crossing in the devices’ current–voltage (I–V) characteristics due to the nanobattery effect or resistance, capacitive, and inductive effects. The device also displayed a negative differential resistance (NDR) effect. Non-volatile storage was feasible with non-zero crossing due to the exhibition of resistive switching behavior. The sweeping range was −10 V to +10 V. These devices had two distinct states: ‘ON’ and ‘OFF’. The ON/OFF ratios of the devices were 14 and 100 under stable operating conditions. The open-circuit voltages (Voc) and short-circuit currents (Isc) corresponding to memristor operation were explained. The DC endurance was stable. Ohmic conduction and direct tunneling mechanisms with traps explained the charge transport model governing the resistive switching behavior. This work gives insight into data storage in terms of a new conception of electronic devices based on facile and low-temperature processed material composites for emerging computational devices.

## 1. Introduction

In the era of the More-than-Moore paradigm [1] and the Internet of Things (IoT), there is a requirement for new materials, device architectures, and technology. This is due to demands for portability, lightweight properties, bendability, stretchability, and ubiquitous applications. This demand evinces interest in flexible electronics. This has led to the development of conformal electronics for health care [2,3], flexible storage systems [3], display systems [4,5], and radio frequency identification systems [6,7]. One of the crucial things to realize about IoT gadgets is that they store information in non-volatile memory. Resistive memory devices [7] have become immensely popular among other emerging non-volatile-memory-like spin-transfer torque magneto-resistive (STT-MRAM) [8] and phase-change RAM (PCRAM) devices [9] over the past few decades. These devices have the advantages of being non-volatile, having fast access times, and having low power consumption [10,11]. Initially, resistive devices used chalcogenides and oxides [12]. With the saturation of the Moore model, there is now demand for new materials. A comparison of various emerging non-volatile memories is provided in Table 1.

Organic materials have provided another alternative for resistive switching memories. They are usually lightweight and flexible and have high data storage capacities and well-defined, simple layered structures making them suitable for resistive memory devices. Organic materials containing conjugated molecules and polymers have been researched for resistive switching memory applications. Small-component organic molecules like boron-based molecules (BF2BTDT) [13], pyrene-fused large N-heterocene (PyTTQ) [14], and benzothiadiazole-based molecules (NONIBTDT) [15] with structural tunability have shown resistive switching properties. In addition to single molecules, mixtures of organic molecules with two or three components have resulted in resistive switching behavior in MIM-type ITO/TCz:PDI/Al devices with tunable properties [16], and mixing a donor of indolo [3,2-b] carbazole (ICz) with a PDI acceptor (ICz:PDI = 2:1, 1:1, and 1:2) has resulted in binary to ternary properties [17]. Bozano and Yang et al. [18,19] have conducted a lot of work on incorporating metallic NPs into organic molecules to achieve the tunable characteristics of bistable memory. Potentially applicable metals are Al, Ag, Mg, Cr, and Ni [18]. They have shown an ON/OFF ratio of >10^4^, a high switching speed of <10 ns (delay), a switching endurance of >10^6^ cycles, and data retention >10^5^ s in multilevel switching devices. Single-component and multiple-layer polymers have optimized the macromolecule structure for bistable and multilevel resistive switching (RS). Some of the single-component materials include PVA composite [20], P55 [21], PMIDO3-based memory devices [22], and PFTPA-Fc [23]. They exhibit RS mechanisms via donor–acceptor (DA) charge transfer and conformational change mechanisms. Polymer blends of poly(methyl methacrylate) (PMMA) and poly(3-butylthiophene) (P3BT), serving as the active component [24], have been used. A Metal–Insulator–Metal (MIM)-type memory structure based on the st-PMMA/C60 complex has shown WORM-type switching characteristics [25].

Here, we are reporting the synthesis of trans, trans-1,4-bis-(2-(2-naphthyl)-2-(butoxycarbonyl)-vinyl) benzene (in short, 2-NVB) [26] and PVA composite as an active material for the resistive switching device. The I–V characteristics exhibited non-zero-crossing, nanobattery, capacitive, resistive, and inductive effects. The charge transport mechanisms and the process of the transfer of carriers between the interface and Ag ions were explored. A sandwich configuration of the M-I-M (Metal–Insulator–Metal) structure was considered. We were looking for an organic material for use as a dielectric for resistive memory devices. An analysis of the behavior was conducted for various transport models.

## 2. Materials and Methods

### 2.1. Materials

In the synthesis process, chemicals like acetone, ethanol, Polyvinyl Alcohol (PVA, India), and hydrochloric acid (HCl, 36.5%, India) were procured from Sigma Aldrich and Merck (India). De-ionized water (resistivity: 18.2 MΩ) was obtained from a Millipore system (India).

### 2.2. Synthesis of PVA Solutions and PVA-2-NVB Composites

A PVA dielectric layer was synthesized by dissolving 0.4 g of PVA powder in 6 mL of absolute ethanol and 4 mL of de-ionized water in a beaker. The beaker with the solution was then stirred on a magnetic stirrer at 120 °C for approximately 3 h until a uniform solution was obtained. 

**Figure 1:**
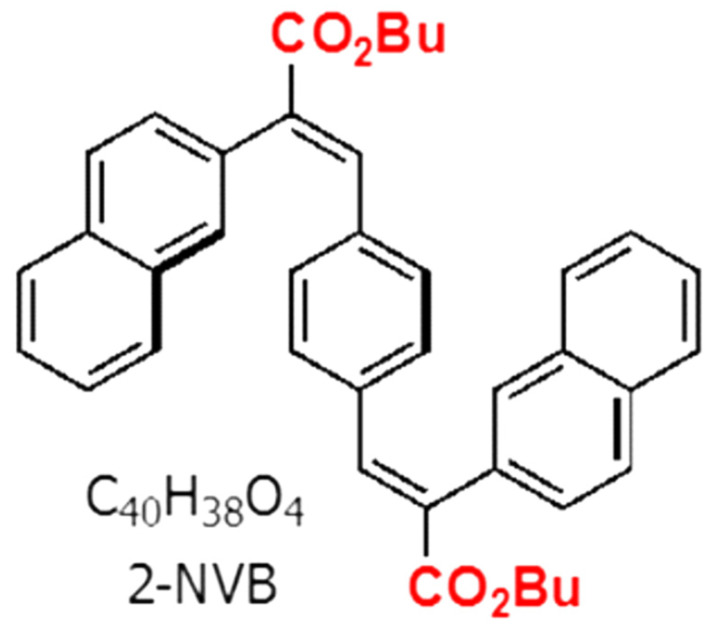
Chemical structure of trans, trans-1,4-bis-(2-(2-naphthyl)-2-(butoxycarbonyl)-vinyl) benzene.

The organic compound used in this work was trans, trans-1,4-bis-(2-(2-naphthyl)-2-(butoxycarbonyl)-vinyl) benzene (2-NVB), which was mentioned in Reference [26]. The molecule had two naphthalene units connected with a central benzene via an ethylene bridge and was thus fully conjugated. Two butyl ester groups were also attached to the molecule for solution processing to change/manipulate the electronic nature of the compound. The compound was a white powder that formed a colorless solvent solution. The chemical structure is shown in Figure 1.

Two samples were prepared for material characterizations: PVA and 1:4::2-NVB: PVA. The synthesis of composite solutions (2-NVB:PVA) is discussed here. The sample was prepared by taking 25% of the compound. A ratio of 0.1 g/0.4 g (2-NVB/PVA) was added to the above solution. It was stirred on a magnetic stirrer until it became homogeneous. 

### 2.3. Device Fabrication

**Figure 2:**
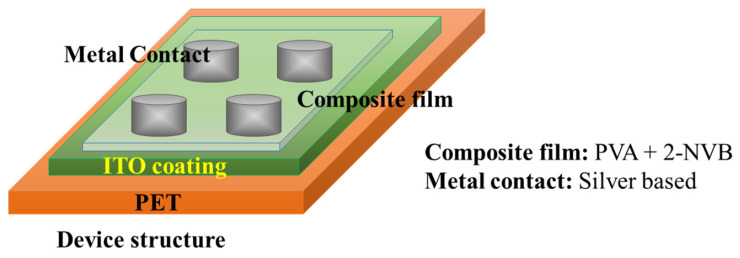
Schematic of M-I-M structure used for this study. The top contact is silver, and the bottom is ITO-coated PET.

Sandwich device structures of Metal/1:4::2-NVB-PVA-/ITO-PET (Metal–Insulator–Metal) and Metal/PVA-/ITO-PET were fabricated to realize the resistive switching phenomenon. The ITO-PET substrate was flexible. A schematic of the fabrication of the MIM structure is shown in Figure 2.

### 2.4. Electrical and Physical Measurements

Electrical characterization was carried out on the M-I-M structure. An M-I-M structure with ITO-coated PET as the bottom contact and a silver material as the top contact was used. The current–voltage (I–V) measurements were carried out using a Keithley 2636 B source meter (Tektronix, Beaverton, OR, USA) in a dark environment at room temperature. A contact area of 0.5 mm × 0.5 mm was used for measuring the resistive switching characteristics. The compliance limit was set to 1 mA to prevent overshooting of the current. The voltages were swept between −10 V and 10 V and vice versa with a voltage step size of 0.1 V. For each swept value of the voltages, currents were measured using the Keithley source meter. The recorded current values were analyzed to establish the various transport models described in the succeeding sections.

The following procedure was used for measurement:

1. The voltages were swept from 0 V toward 10 V, and currents were measured.
2. Initially, the current was very small (high resistance), and it increased toward 10 V.
3. At +10 V, the device switched to the low-resistance state (LRS). After that, voltages were swept from 10 V to −10 V, and the output current decreased and then increased toward −10 V.
4. Afterwards, voltages were swept from −10 V toward 0 V. At −10 V, the current started decreasing toward 0 V. It was at the minimum value (high-resistance state).

The X-ray diffraction (XRD) spectra of the films were measured using a Miniflex 600 version Rigaku diffractometer (Rigaku, Tokyo, Japan) with Cu Kα radiation with λ = 1.54 Å. Thermal studies of the electrolyte were conducted using a differential scanning calorimeter (DSC-60Plus, SHIMADZU, Kyoto, Japan) equipped with a thermal analysis data station.

## 3. Results

### 3.1. Structural and Thermogravimetric Analysis

**Figure 3:**
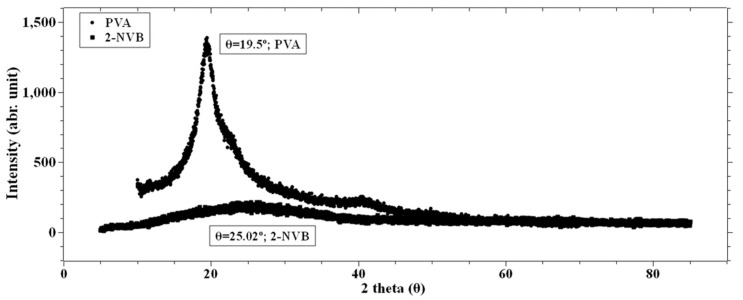
XRD spectra of PVA and 2-NVB. PVA film shows a peak at 19.49° with a sharp shoulder and a low broad peak at 38.6°.

Figure 3 shows the X-ray diffraction patterns of pure PVA and pure 2-NVB. It can be observed from Figure 3 that PVA film exhibits semi-crystallinity [27,28], showing a relatively sharp peak at 19.49° with a shoulder and low-intensity broadening at 38.6°. This indicates that the peak corresponds to an orthorhombic lattice, indicating a semi-crystalline nature. The internal structure consists of both intramolecular and intermolecular hydrogen bonds. Molecules in an individual monomer unit or even in different monomer units have these types of bonds [29]. Figure 3 also shows the spectrum of 2-NVB. It shows a broad peak at 24.8°, indicating it is not crystalline. 

**Figure 4:**
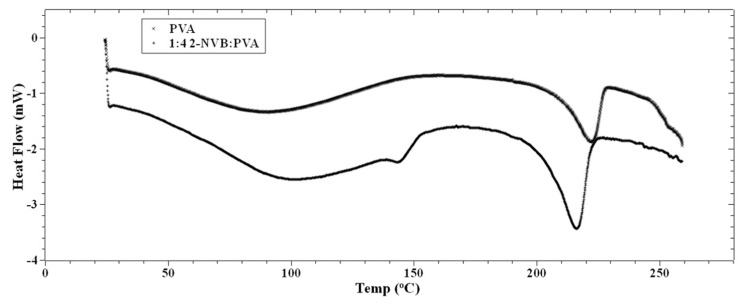
DSC thermographs of PVA and 1:4 2-NVB:PVA. Differential scanning calorimetry exhibits the materials’ phase transition temperatures. The PVA’s glass transition temperature is 90.41 °C, and for 2-NVA this value is 105.44 °C.

The crystallite size can be calculated using the Scherrer formula given by

(1) $$D=\left(\frac{0.9\lambda }{\beta \cos\theta }\right)$$

where *λ* is X-ray wavelength (1.54 Å), *β* is the full width at half the maximum of the diffraction peak, and *θ* is the diffraction angle [30]. The effect of increasing the concentration of 2-NVB in PVA on the thermal properties of the composite layer was studied using Differential Scanning Calorimetry (DSC), as shown in Figure 4. This provided a better understanding of the intermolecular interactions between 2-NVB and PVA. The DSC graphs for pure PVA and 1:4::2-NVB:PVA are shown in Figure 4, and the data are enumerated in Table 2.

The PVA film’s glass transition temperature (Tg) was 90.41 °C, whereas, for 1:4::2-NVB: PVA, this value was 105.44 °C. Hence, we can note that increasing the concentration of 2-NVB in PVA caused an increase in the glass transition temperature for the 1:4::2-NVB: PVA sample. The increase in the first case could be due to the formation of H/OH bonds between 2-NVB and PVA molecules in the matrix. A similar study carried out by Shokralla et al. [31] on the thermal properties of an epoxy/phenolic resin (NOVOLAC) composite demonstrated that H bonding is the main factor for the increase in the glass transition temperature of the composite. This proves that increasing the concentration of 2-NVB in PVA improves thermal rigidity. An endothermic process occurs at 213.19 °C with a melting point of 222.23 °C. The slight reduction in temperatures for the endothermic processes and melting points indicates increased crystallinity of the polymer matrix.

### 3.2. Electrical Characterization: Basic Analysis of I–V Characteristic

**Figure 5:**
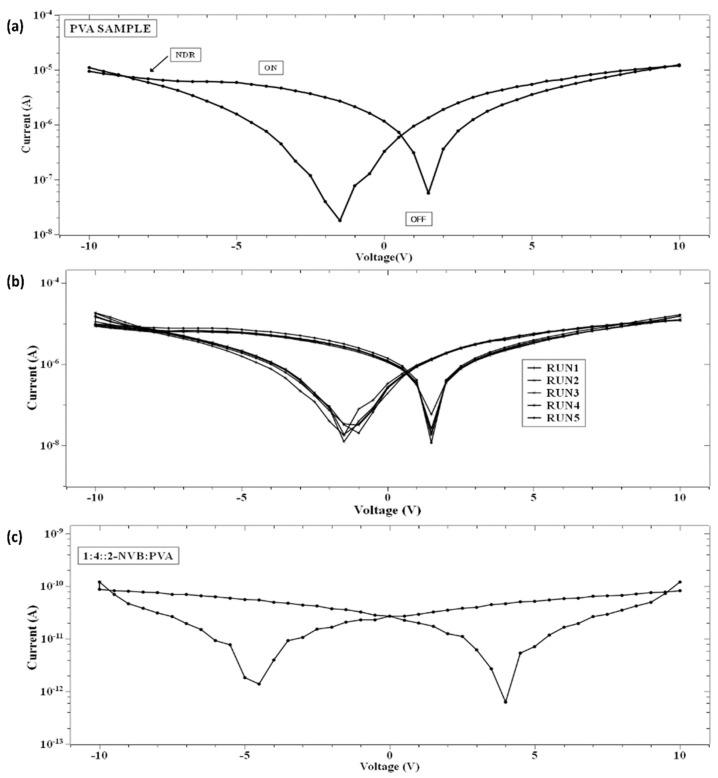
I–V Characteristics of the PVA sample for (**a**) a single run, (**b**) multiple runs under normal conditions, and (**c**) 1:4::2-NVB:PVA. I–V curves exhibit the switching characteristic in PVA and PVA:NVB composite films. Multiple runs demonstrate the repeatability and stability of the films.

Insight into current–voltage (I–V) characteristics will highlight device switching behavior. Figure 5a shows the current–voltage (I–V) characteristic curves of the simple PVA resistive switching memory devices, and their multi-run characteristic is shown in Figure 5b. First, voltage scans with different voltage ranges were analyzed. Four cases were considered. The DC voltage was swept from 0 V to 10 V, from 10 V to −10 V, and from −10 V to 0 V. In other words, each sweep was carried out as follows: 0 V→10 V→ 0 V→−10 V→0 V. The voltage sweep rate was 500 mV/s, and a current compliance of 0.01 mA was applied. The bottom contact was the ITO-PET sheet, and the top contacts were made out of a silver contact with a positive bias. The I–V curve showed bipolar switching characteristics. The same procedure was repeated for all samples (Figure 5a–c). Figure 5c shows the current–voltage (I–V) characteristic curves of 1:4::2-NVB: PVA-based composite films, which also show the switching characteristic. The sample (PVA) was initially in a high-resistance state (HRS) while sweeping the voltage from 0 V to +10 V. It was observed that it changed its value from ~10^−7^ to ~10^−5^ A. This was a switching operation of the device, with the state changing from a high-resistance state (OFF) to a low-resistance (ON) state. The transition from the OFF state to the ON state was like a “writing” process in a memory device. Upon reverse sweeping, the ON state was reverted to the initial OFF state. This was like an “erasing” process. It was the OFF state of the device. In this way, the cycle of “write-read-erase-read” (WRER) could be achieved for a non-volatile rewritable memory device. This was a repeatable process. After that, it changed to a low-resistance state (LRS). It was kept for some time and again changed to a high-resistance state (HRS). The current decreased by nearly two orders while sweeping in the reverse direction from +10 V to 0 V. Then, during negative biasing from 0 V to −10 V, the current increased nearly two-fold, and the cycle repeated. This was also true for 1:4::2-NVB: PVA-based composite films (Figure 5c). The composite materials’ properties were stable and steady. The ON/OFF ratios of the devices were 14 and 100, and stable operation was found. The DC endurance ratio showed promising results.

### 3.3. Analysis of Hysteresis in the I–V Characteristic

**Figure 6:**
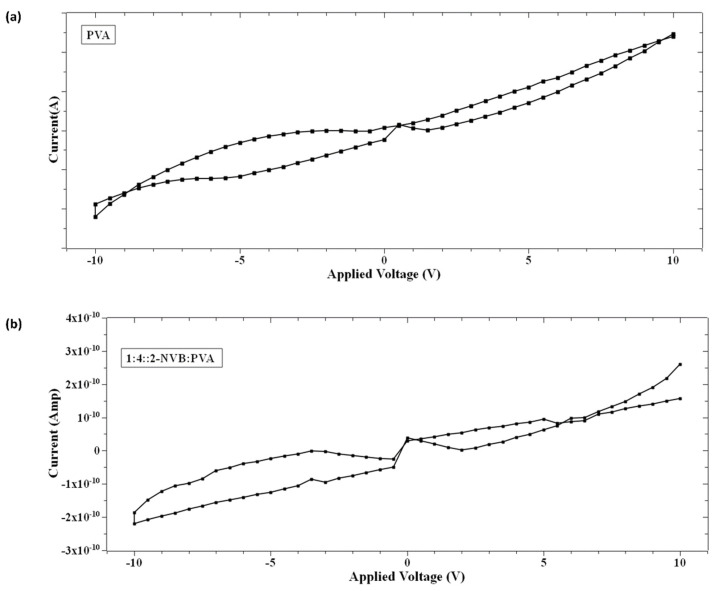
Hysteresis loop in the I–V characteristic of (**a**) PVA sample and (**b**) 1:4::2-NVB:PVA. It shows the devices may belong to the memristor class.

On further analysis of the I–V characteristics, it was observed that the I–V curves show some butterfly shapes and non-zero current minima (the minimum current is not at 0 V). Typically, this is an indicator of charge trapping/de-trapping phenomena and the generation of an internal field due to carriers. It is also observed in Figure 5 that the minimum value of current does not appear at zero V as in other memory devices with zero-crossing hysteretic curves. The current values (minimum values) differ, indicating that the sample’s resistivity varies. That means that at zero V, the device retains some charges. This could be due to either the nanobattery effect [32] or the co-existence of capacitive, resistance, and inductive effects [33]. This could be due to structural rearrangement during this type of applied voltage and the orientation of charge carriers [33,34,35]. Majumdar et al. [36] suggested that a charge stored in the conjugated polymer near the electrode/polymer interface is the main contributor to charge injection and, hence, the non-zero minima in the I–V characteristic [36]. Terms like Voc (open-circuit voltage, a non-zero voltage at zero current) [36] and Isc (short-circuit current, a non-zero current at zero voltage) [36] were defined to quantify the parameters to explain the origin of this characteristic. Figure 6a,b show the hysteresis curves in our samples. As explained by [37], the origin of the hysteresis characteristic is due to the presence of a displacement current (*I_d_*) adding to the conductive part (*I_r_*). *I_d_* is given by

(2) $$I_{d}=\frac{d\left(CV\right)}{dt}=C\times \frac{d\left(V\right)}{dt}+V\times \frac{d\left(C\right)}{dt}$$

The contribution of *I_d_* is due to voltage variation (first term) and capacitance variation (second term). The total current (*I*) is given by

(3) $$I=I_{d}+I_{r}$$

In the case of our samples, mechanisms proposed by Majumdar et al. [36] were considered. Initially, a voltage sweep was performed from 0 V to a positive voltage (small to mid-level). At this time, space charge carriers accumulated in the films (PVA or 1:4::2-NVB:PVA) near the ITO electrodes, and opposite carriers collected near the Ag electrodes. With a further increase in the voltage sweep, space charge distribution occurred along the complete lengths of the films. Then, while traversing from a positive voltage to 0 V, this distribution of charge carriers remained, creating a potential barrier. A small positive voltage called an open-circuit voltage (+Voc) was required to offset this potential and maintain the current at zero. At zero voltage, current flowed due to charge carriers in the external circuit, leading to a negative short-circuit current (−Isc). Typical values of +Voc and −Isc for our samples are enumerated in Table 3. In the next cycle, charge storage happened while traversing from 0 V to a negative voltage. In subsequent sweeping from a negative voltage to 0 V, negative (−Voc) and positive (Isc) currents were obtained. The significant negative voltage was due to the long discharge time, and this has been observed in bio-memristors [37,38]. This negative cycle was the same as the positive cycle discussed above. Typical values of −Voc and +Isc for our samples are enumerated in Table 3. Different values could be due to the work function difference of Ag and ITO. Similar non-zero hysteresis behaviors have been elucidated for several conjugated polymers [36], biomaterials [39], and multiferroic BiFeO_3_ nanoribbons [40] in non-zero-crossing-based memristive systems and the references in [38].

### 3.4. Analysis of Capacitive Effect and Negative Differential Resistance (NDR) 

**Figure 7:**
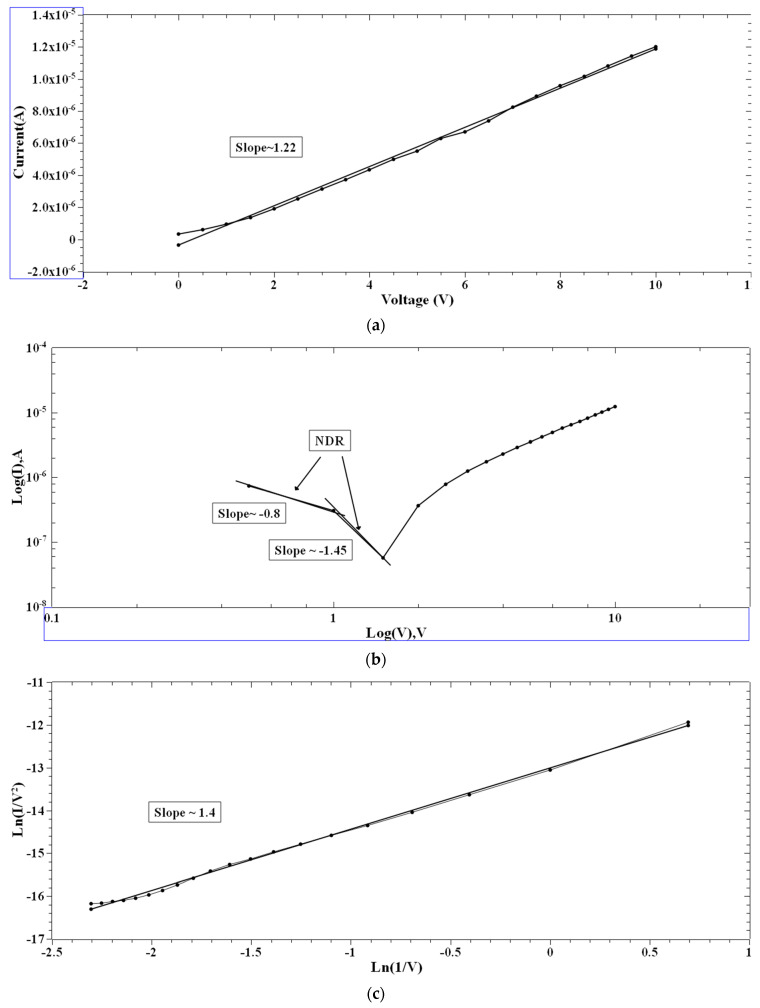

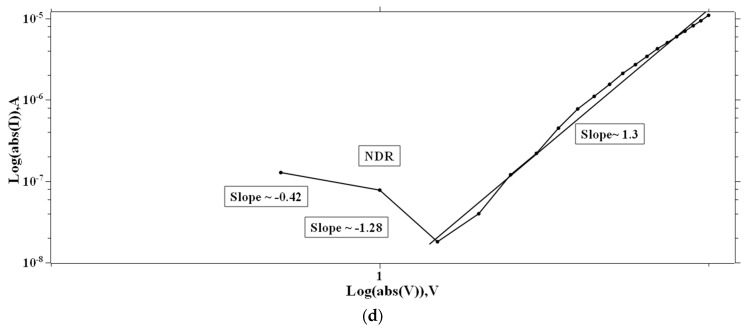
The fitted I–V hysteresis curves of Ag/PVA/ITO-PET: (**a**) I–V fitting, (**b**) Log(I) vs. Log(V) fitting, (**c**) Ln(I/V^2^) vs. Ln(1/V) characteristic plot, and (**d**) Log(abs(I)) vs. Log(abs(V)) plot.

**Figure 8:**
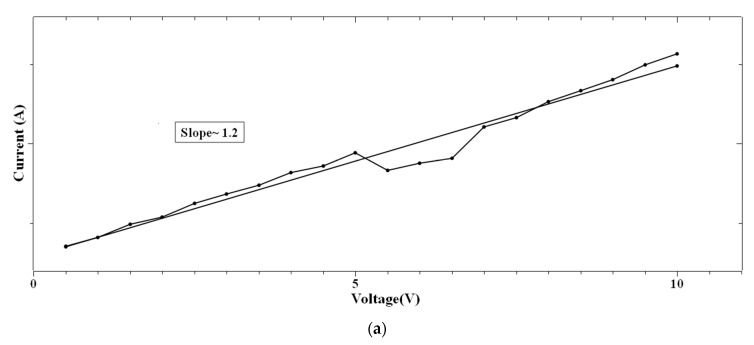

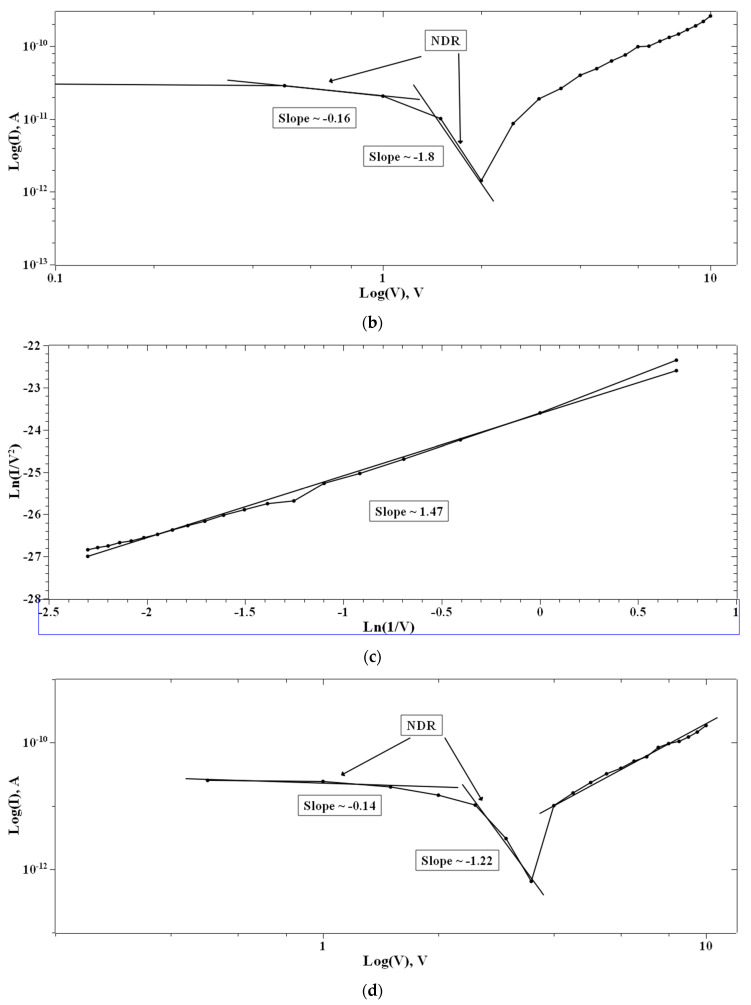
The fitted I–V hysteresis curves of Ag/1:4::2-NVB:PVA/ITO-PET: (**a**) (I)–(V) fitting, (**b**) Log(I) vs. Log(V) fitting, (**c**) I–V characteristic plot, and (**d**) Log(abs(I)) vs. Log(abs(V)) plot.

The I–V characteristic also shows a resistance-capacitive effect in memory devices [38]. This capacitive effect is due to the aggregation of carriers at the interface between an electrode and a composite material. Upon applying the applied bias, carriers (+v and –ve) will move in the opposite direction and form a conductive electrode by shorting the top and bottom parts. Also, a non-linear phenomenon of negative differential resistance (NDR) [41] was observed in the pure PVA devices and other samples (in a limited voltage range), as shown in Figure 7 and Figure 8, along with the resistive switching phenomenon. In normal conditions of NDR, electron density increases with electric fields. But in some devices, an abrupt scenario of a decreasing current with an increasing field can happen, which is NDR. This is also exhibited by graphene nanoribbon (GNR) junctions after a tunnel gap is introduced [41], Pt/CeO2-x/Pt resistive memory devices [42], Cysteine-Functionalized WS2 Quantum Dots [43], Continuous Film Based on Zeolitic Imidazole Framework-8 [44], and organic poly (3, 4-ethylenedioxythio phene): Poly (styrenesulfonate) film [45].

### 3.5. Possible Charge Transport Mechanisms in the Resistive Device

As discussed earlier, the conduction mechanisms responsible for switching behavior were investigated by analyzing the I–V characteristic in the full DC range. The analysis of the charge transport mechanisms enabled a better understanding of the switching window of the memory device. In the MIM structure, two types of conduction mechanisms have been studied: (i) metal-interface-based Fowler–Nordheim (F–N) tunneling and thermionic field emission; (ii) bulk-limit-based Schottky emission, hopping conduction, direct tunneling, and Ohmic conduction [46,47].

The Schottky emission model is governed by

(4) $$I=\frac{4\pi qm^{*}{\left(kT\right)}^{2}}{h^{3}}\exp\left[\frac{-q(\phi -\sqrt{qV/4\pi \varepsilon }}{kT}\right]$$

where *q* is the electron charge, m* is the electron effective mass, *k* is Boltzmann’s constant, *T* is the absolute temperature, *h* is Planck’s constant, *ϕ* is the junction barrier height, *V* is the electric field, and *ε* is the permittivity. When the temperature is constant, the Schottky emission model is approximated by

(5) $$\begin{array}{l} I\alpha \exp\left[\frac{\beta }{kT}\sqrt{V}-\frac{\phi }{kT}\right]\\ \beta =\sqrt{q^{3}/4\pi \varepsilon } \end{array}$$

where *β* is constant and the fitted relationship of ln(*I*) *α* can determine the Schottky emission model dominated by injections of charge carriers. In addition, hopping conduction (simple model) is likely to be represented by

(6) $$I=V\exp\left[\frac{-Ea}{kT}\right]$$

where *E_a_* is the activation energy, *k* is Boltzmann’s constant, and *T* is the temperature in Kelvin. Direct tunneling is modeled by

(7) $$I=V\exp\left[\frac{-2d\sqrt{2m\phi }}{h/2\pi }\right]$$

where *h* is the Planck constant and *d* is the thickness of a film. A logarithmic scale is used for fitting purposes.

The following model is approximated as

(8) $$\ln\left(\frac{I}{V^{2}}\right)\alpha \ln\left(\frac{1}{V}\right)-\frac{2d\sqrt{2m\phi }}{h/2\pi }$$

The above equation points to the voltage dependency of the direct tunneling of charge carriers. The Ohmic conduction of the I–V characteristic curve is modeled by

(9) $$I=\sigma V=q\mu N_{c}V\exp\left[\frac{-\left(E_{c}-E_{F}\right)}{kT}\right]$$

where *σ* is the conductivity, *µ* is the electron mobility, *N_c_* is the effective density of states, *E_C_* is the conduction band edge, and *E_F_* is the Fermi level.

Single and multiple fits were carried out for the I–V characteristics of the PVA and 2-NVB-PVA samples to determine possible transport mechanisms. Figure 7a–d illustrate the charge transport model for the PVA case in four different regions. In Figure 7a, a fitting model was applied to the I–V characteristic. It was fit with a linear-fit model in the double logarithmic scale. It shows Ohmic conduction, and the slope of the fit is ~1.22. In the biased region, several thermally generated carriers in the composite film far exceed the charge carrier injection from the electrode. This shows the formation of the conducting filaments in the device. The slope is not ~1 but ~1.22 due to the carriers’ drifting due to the high applied voltage. Similarly, Figure 7b,d show I–V characteristics that can be fit with Log(I) vs. Log(V), showing the NDR effect (slope of negative linear fit) and Ohmic conduction behavior (slope of positive linear fit). In Figure 7c, experimental data were linearly fit with Ln(I/V2) vs. Ln(1/V), which might be due to direct tunneling [47]. Tunneling happened through the trapped sites, overcoming the barriers due to the accumulation of charge carriers at the interface. However, extensive XPS, FESEM, and TEM studies are required to ascertain this phenomenon. It was the same for the other sample.

In the case of 1:4::2-NVB:PVA (Figure 8a–d), from 0 V to 10 V, Ohmic conduction was observed to follow the simple I–V characteristic. NDR conduction was observed in the range of 10 V to 0 V. In the 0 V to −10 V range, the experimental data were linearly fit with Ln (I/V2) vs. Ln (1/V) tunneling with trap states. From −10 V to 0 V, NDR and Ohmic mechanisms were exhibited. Table 4 lists some resistive switching device parameters of the same types of materials. It is observed that all the materials have high sweeping windows, small ON/OFF ratios, and bipolar devices. However, some devices have good ON/OFF ratios. Our device also exhibits a high sweeping window and a maximum ON/OFF ratio of ~10^2^. This value is less than the threshold of ~10^4^. However, this is our initial study, and optimized work will be a part of future studies.

### 3.6. Possible Model for Resistive Switching Device

A possible mechanism is discussed here. PVA is a hydrophilic linear polymer with good mechanical and thermal stability [60]. This facilitates ionic conduction in the matrix. Because of this, it shows switching behavior. The link between the top and bottom electrodes indicates the onset of the set process, possibly due to electrochemical metallization and Ag filament formation under an electric field [61]. The device structure comprises an active electrode (Ag) and an ion-conducting insulating material. Authors [61] have observed the formation of Ag bridges and dissolution in polymer cells. During the application of the positive bias, the Ag top electrode takes the form of Ag+ ions and the liberation of electrons. The Ag+ ions diffuse through the matrix of the PVA-2-NVB sample. From the bottom electrode, electrons travel toward the top part and form a bridge with the top part, thereby switching the LRS. PVA matrix has abundant hydroxyl groups that can attach to carbon groups, and 2-NVB has nanographene molecules with helix-like structures. These molecules have seven benzene rings [26]. DFT calculations predict the helicity in such doubly helical carbon nanostructures [26]. In addition, 2-NVB has π-extended molecules that show enhanced conjugation. PVA has a delocalized π-system backbone along polymeric chains. Trapped electrons reduce the Ag+ ions’ travel speed in the composite or ITO. When a positive bias is applied and it reaches the boundary of the turn-on voltage, a filament is formed in the composite. It increases the current and sets the device in the ON state. Upon changing the polarity of the bias of the top electrode, joule heating can disrupt the link, and the device can change to an OFF state due to the rupturing of the link. Thus, the ON–OFF ratio is two orders higher than in the case of PVA alone. The conductivity is enhanced due to sp^2^ bonds in the main structure.

## 4. Conclusions

In summary, we investigated the electrical characteristics of PVA film and a newly synthesized trans, trans-1,4-bis-(2-(2-naphthyl)-2-(butoxycarbonyl)-vinyl) benzene-based aryl acrylate (2-NVB)-PVA composite for non-volatile storage applications. They exhibited both resistive switching and the NDR effect. An investigation of the I–V characteristic displayed a hysteresis loop with non-zero crossing, showing our devices’ capacitive nature (extended memristor). They had good ON/OFF ratios of 14 and 100, with good endurance and retention properties. The charge transport model conformed to Ohmic conduction and filament conduction in the different regions of operation. Our work attempts to use a facile processed composite for flexible, foldable, and large-area applications.

## Figures and Tables

**Table 1:** Comparative analysis of various emerging non-volatile memories.

Type	Advantages	Disadvantages
ReRAM	- Simple architecture - High device density - Composed of simple and versatile materials	- Forming voltage - High write latency, sneak path - Non-uniformity
PCM	- Well established - Stable performance	- Noise and reliability issues - Large power requirement while switching
STTRAM	- Good performance - Novel mechanisms - Well-established phenomenon	- Power stability issues - Write failure and read disturb
FeFET	- Simple one-transistor structure - Good endurance and scalability - Low field requirements	- Short memory retention - Suffers from charge trapping - Depolarization field

**Table 2:** DSC temperature data of PVA and 2-NVB:PVA.

Compound	Glass Transition Temperature (Tg) (°C)	Onset Temperature (To) (°C)	Endset Temperature (Te) (°C)	Melting Point (°C)	Enthalpy (J/g)
PVA	90.41	213.19	227.11	222.23	−47.24
1:4::2-NVB:PVA	105.44	209.22	223.57	218.77	−35.75

**Table 3:** Enumerated values of open-circuit voltages (Voc) and short-circuit currents (Isc) in the case of PVA and 2-NVB-PVA composite.

Sample Name	Type of Applied Voltage	Voc (V)	Isc
PVA	Positive biasing	+1	−1.3 μA
	Negative biasing	−1.06	0.33 μA
1:4:: 2-NVB:PVA	Positive biasing	1.5	−52.5 pA
	Negative biasing	−3.4	23.67 pA

**Table 4:** Comparative analysis of resistive switching memories.

Structure	Deposition Technique	ON/OFF Ratio	Sweeping Window	No. of States	Ref.
ITO/P3HT:4CzIPN or 2CzPN/Al	Spin coating	10^5^	−8 V to 8 V	2	[48]
Ag/keratin/ITO	Solution processing	~10^4^	−6 V to +6 V	2	[49]
Ag/ZnO/PVA:MoS_2_/ITO	Sputtering, spin coating	~10^4^	−3 V to +3 V	2	[50]
Al/Au NPs:lignin/Al	Spin coating	~10^4^	−6 V to 5 V	2	[51]
Al/silk fibroin/ITO	Spin coating	~1	−15 V to 15 V	2	[52]
Al/gelatin/ITO	Spin coating	~10^4^	−6 V to +6 V	2	[53]
Au/Ni/FeOx-GO/Si_3_N_4_/n^+^-Si	PECVD, spin coating	~10^4^	−10 V to 10 V	2	[54]
ITO/PMMA:MWCNT-COOH/Ni	Spin coating	~10^7^	−6 V to +6 V	3	[55]
Ag/Bphen/MAPbBr3/PEDOT:PSS/ITO	Spin coating	80	0 V to +6 V	1	[56]
Ti/TiO2/CH3NH3Pb ClXI3-X/Au	Coating	20	−5 V to +5 V	2	[57]
Al/Cs 3Cu 2I5/ITO	Spin coating	65	−1 V to 1 V	2	[58]
ITO/PEI/CH_3_NH_3_PbI_3_/PEI/metal	Coating	20	−1 V to 1 V	2	[59]
Ag/2-NVB-PVA/ITO	Spin coating	~10^2^	−10 V to +10 V	2	Present work

## Data Availability

The data are available from the corresponding author on request.
